# Impact of COVID-19 Pandemic on Non-Small Cell Lung Cancer Care

**DOI:** 10.3390/curroncol30010059

**Published:** 2023-01-06

**Authors:** YiYuan Zhai, Pooja Chopra, David Kang, Nicholas J. Robert, Wei Zhang

**Affiliations:** Ontada, 6555 State Highway 161, Irving, TX 75039, USA

**Keywords:** COVID-19 impact on cancer care, non-small cell lung cancer, immunotherapy dosing schedule, cancer treatment delay, cancer screening delay, cancer diagnosis delay

## Abstract

We assessed the impact of COVID-19 on healthcare visits, timing of stage IV NSCLC diagnosis and immunotherapy initiation, and rates of switching to extended dosing schedules of immunotherapies among patients with stage IV NSCLC. This retrospective study examined electronic health record data of adult patients receiving treatment for stage IV NSCLC within The US Oncology Network and Onmark. Endpoints were compared for February–July 2019 (before COVID) vs. February–July 2020 (during COVID). The study found rapid decreases in numbers of patients with clinic/vital visits, immunotherapy initiations, and new diagnoses of stage IV NSCLC during April–May 2020 vs. April–May 2019. The rate of delays of immunotherapy administrations and proportions of patients with such delays increased from February to March of 2020. These patterns may have resulted from the increase in COVID-19 cases during this period and the corresponding quarantine and lockdowns. However, when comparing pre COVID-19 and during COVID-19 for May and after, the differences in delay of immuno-oncology administrations became less marked, likely due to lifting of lockdowns. The rate of switching from shorter to longer dosing schedules increased from May–July 2020. This was mainly attributed to pembrolizumab, likely due to FDA approval of the pembrolizumab 6W dosing schedule in April 2020.

## 1. Introduction

The COVID-19 pandemic has had a significant and detrimental impact on healthcare delivery in the United States. The ongoing crisis has presented multiple challenges for cancer care delivery and has adversely impacted cancer diagnosis, patient encounters, and management of treatment [1].

According to 2019 data from the National Cancer Institute’s Surveillance, Epidemiology and End Results Program, approximately 50% of patients with non-small cell lung cancer (NSCLC) are diagnosed with distant stage disease [2]. Some studies have suggested that patients with cancer in addition to COVID-19 are at higher risk for severe complications or death than those without cancer, particularly those with lung cancer [3,4,5,6,7]. Patients with cancer would already be more likely to contract COVID-19 illness, given their immunocompromised state owing to the tumor microenvironment and treatments such as chemotherapy [7,8]. With the high transmissibility and morbidity of COVID-19 and the associated demand on healthcare resources, the American Cancer Society, the American Society of Clinical Oncology, and the Centers for Medicare and Medicaid Services have recommended postponement of cancer screening procedures that require clinic visits in order to conserve health system resources and protect patients from exposure to COVID-19 [3,9,10]. With the changes in resource availability and patient-related factors, delays have been observed in cancer screening, diagnosis, and treatment [11,12]. These delays in turn may account for observations of short-term decreases in cancer diagnosis rates, increased diagnoses of more advanced cancer, and suboptimal treatment patterns [3,4,13,14,15,16].

Within this challenging environment, the goal is to ensure that patients with life-threatening late-stage cancer still receive appropriate and timely treatment. Limited studies have been conducted to quantify the immediate impact of COVID-19 on normal cancer care activities for patients with stage IV NSCLC in a real-world setting. In a prospective cohort study of patients with outpatient visits in March 2020, patients with lung cancer were more likely to have delays in diagnostic procedures and treatment compared with other solid tumors; approximately two thirds of those patients had metastatic cancer and two thirds were receiving systemic therapy at index, including approximately 40% receiving ICI [11].

Immunotherapy has been associated with promising results among patients with metastatic NSCLC. Until recently, immunotherapy regimens have required a high frequency of infusion visits (i.e., every 2 or 3 weeks) [17,18,19]. However, the FDA within the last few years has approved longer dosing schedules for several immunotherapies—atezolizumab (approval 18 May 2020, 1680 mg every 4 weeks), nivolumab (approval April 2018, 480 mg every 4 weeks), and pembrolizumab (accelerated approval 28 April 2020, 400 mg every 6 weeks)—for metastatic NSCLC [20,21,22]. These schedules could help patients reduce clinical visit frequency and at the same time yield similar efficacy and safety results [15,23,24]. Various articles and sets of guidelines have suggested modifying or expanding immunotherapy treatment intervals to reduce clinical visit frequency and duration during the pandemic [15,24,25].

In this study, we evaluated the patterns of immunotherapy treatment among patients with stage IV NSCLC before and during the COVID-19 pandemic. Specifically, we assessed the impact of COVID-19 on timing of stage IV NSCLC diagnosis, time to treatment initiation (TTI), incidence of delay in treatment of immunotherapy, and the adoption of extended dosing schedules of immunotherapies and the corresponding switching patterns among patients with stage IV NSCLC.

## 2. Materials and Methods

### 2.1. Study Design and Data Source

This was a retrospective study of adult patients with stage IV NSCLC receiving treatment at practices in The US Oncology Network and Onmark that utilize the iKnowMed (iKM) electronic health record (EHR). iKM is an integrated web-based database of the oncology-specific EHR system maintained by McKesson Life Sciences. iKM captures data on outpatient practice encounter histories as well as demographics (e.g., age, sex), clinical (e.g., cancer diagnosis, disease stage), laboratory results, and treatment information (e.g., line of therapy, administration) for patients within The US Oncology Network and select Onmark sites. More than 2700 oncology providers use the iKM EHR [26]. The US Oncology Network includes 1400 affiliated physicians operating in over 500 sites of care across the United States and treats approximately 1.2 million US patients diagnosed with cancer per year [27]. Data analyzed in the study were obtained through programmatic database abstraction from the iKM EHR.

The study compared patient characteristics and treatment patterns across two timeframes: before (February 2019 to July 2019) and during the COVID-19 pandemic (February 2020 to July 2020). These time periods were based on the COVID-19 timeline defined by the Centers for Disease Control and Prevention. The World Health Organization declared COVID-19 a pandemic on 11 March 2020. US states began to shut down to prevent the spread of COVID-19 on 15 March 2020, and by 13 April 2020, most US states were reporting widespread cases [28].

### 2.2. Patient Population

Patients were included if they had a diagnosis of stage IV NSCLC, were at least 20 years old at diagnosis, and had at least 1 visit within The US Oncology Network or Onmark within the study period. Patients were excluded if they had a diagnosis of early-stage NSCLC, had been enrolled in a clinical trial in their treatment history, or had a diagnosis of another primary cancer.

**Figure 1:**
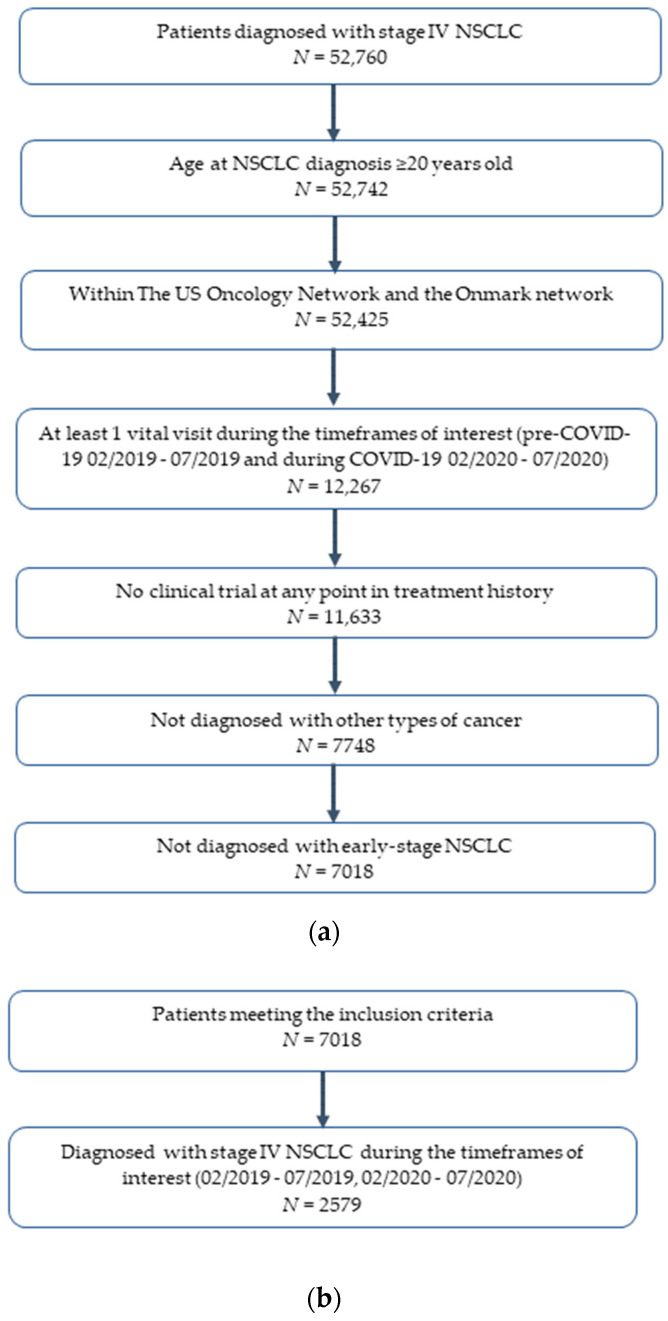

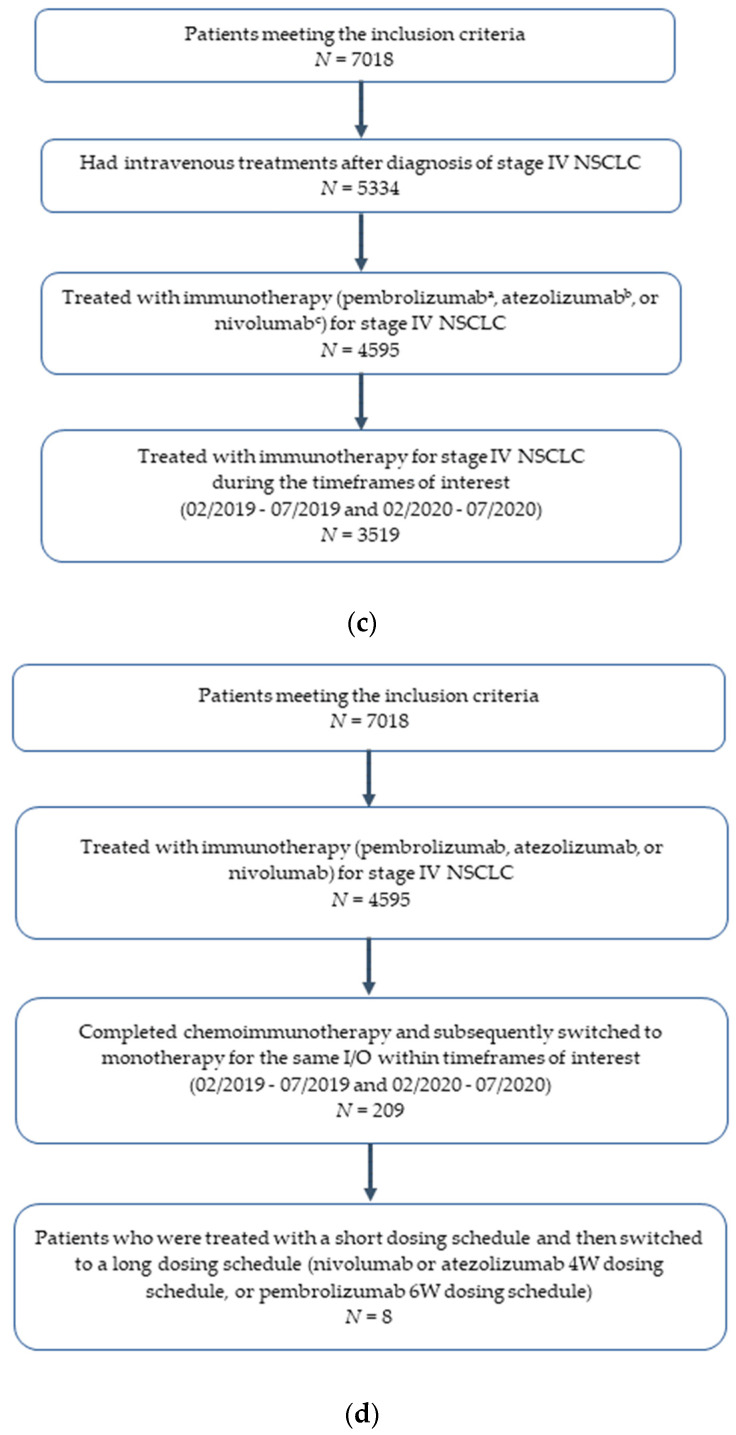
Patient selection flowchart for (**a**) patients meeting the inclusion criteria (**b**) patients with newly diagnosed stage IV NSCLC (**c**) new and continuing immunotherapy-treated patients (**d**) switchers; 4W, once every 4 weeks dosing; 6W, once every 6 weeks dosing; I/O, immuno-oncology agent; NSCLC, non-small cell lung cancer. Recommended dosing schedules for metastatic NSCLC; ^a^ Pembrolizumab 200 mg q3w or 400 mg q6w (FDA approval of longer dosing schedule 28 April 2020) [21]; ^b^ Atezolizumab 840 mg q2w or 1200 mg q3w or 1680 mg q4w (FDA approval of longer dosing schedule 18 May 2020) [20]; ^c^ Nivolumab 240 mg q2w or 480 mg q4w (FDA approval of longer dosing schedule April 2018) [22].

Patients selected for the study were further stratified into three cohorts (Figure 1). Patients in the newly diagnosed stage IV NSCLC cohort had to have received that diagnosis within the timeframes of interest. Patients in the new and continuing immunotherapy cohort had initiated or received at least one administration of an immunotherapy (pembrolizumab, atezolizumab, or nivolumab) during the timeframes of interest. The switcher cohort consisted of patients who previously completed chemoimmunotherapy with a shorter dosing schedule and subsequently switched to monotherapy for the same immuno-oncology agent (I/O) with a longer dosing schedule during the study period.

### 2.3. Statistical Analyses

Descriptive analyses were conducted to assess demographic and clinical characteristics among the overall cohort of patients who fit all the selection criteria, as well as for the subgroups.

#### 2.3.1. Measurement of Diagnosis and Treatment Encounters

To quantify the effects of COVID-19 on patient encounters, the following metrics were generated by month, comparing February to July 2019 data with February to July 2020 data: clinic/vital visits incidence, patients newly diagnosed with stage IV NSCLC, and initiation of immunotherapy.

The point-in-time comparison of monthly trend was performed. Percent change between each month was calculated by subtracting the patient count in 2019 from the same month in 2020 and dividing that by the patient count in 2019.

#### 2.3.2. Measurement of Time to Treatment Initiation (TTI)

Among the subgroup of patients with newly diagnosed stage IV NSCLC, the following criteria were applied. Patients who were not given any treatments after diagnosis were excluded, as were patients whose first treatment occurred >180 days after diagnosis.

TTI was defined as time in days from stage IV diagnosis date to initiation of anti-cancer treatment. The mean and median TTI were compared between the pre-COVID-19 vs. during-COVID-19 cohorts.

Differences in patient characteristics between the pre-COVID-19 and during-COVID-19 cohorts were evaluated using chi-squared tests and independent *t*-tests. The association between timing of diagnosis and TTI was evaluated using a multivariate linear regression model by adjusting for other independent variables (type of treatment, age, gender, race, histology, and Eastern Cooperative Oncology Group performance status (ECOG PS) score).

#### 2.3.3. Measurement of Delay in Administration of Immunotherapy

Patients within the cohort of new and continuing immunotherapy were included in this analysis. In addition, only patients who received flat dose administration were considered.

In the iKM EHR system, a physician can select the intended dosing schedule for a particular regimen. The subsequent infusions are then automatically set up in the flowsheet.

The scheduled time for treatment was determined based on the previous administration date plus the planned dosing schedule based on dosage. For example, if a patient was administered a dose of 240 mg nivolumab on 1 February 2020, the scheduled time for next treatment would be 15 February 2020 (1 February 2020 + 14 days). If the line of therapy was changed, the scheduled time for treatment was recalculated from the initial infusion within the new line.

A delay in treatment was defined as receiving a subsequent treatment ≥5 days after the scheduled time for that treatment. The percentage of incidences of delay in treatment was defined as the number of incidences of delay in immunotherapy/number of incidences of immunotherapy administration. This percentage was compared between the pre- vs. during-COVID-19 cohorts by month.

#### 2.3.4. Measurement of Dosing Schedule Switching Rate

The monthly trend of dosing schedule switching rate for immunotherapies of interest was assessed for the overall study population. Patient dosing schedules were tracked over time for pembrolizumab, atezolizumab, and nivolumab in the stage IV setting. Dosing schedules were determined based on the documented dose information or the gap in days between two consecutive infusions. The documented dose took precedence over the gap rules (Supplementary Table S1). The switching rate was calculated as follows:

$$\mathrm{Switching}\;\mathrm{rate}=\frac{\mathrm{Number}\;\mathrm{of}\;\mathrm{patients}\;\mathrm{who}\;\mathrm{switched}\;\mathrm{from}\;a\;\mathrm{shorter}\;\mathrm{dosing}\;\mathrm{schedule}\;\mathrm{to}\;a\;\mathrm{longer}\;\mathrm{dosing}\;\mathrm{schedule}}{\mathrm{Number}\;\mathrm{of}\;\mathrm{patients}\;\mathrm{who}\;\mathrm{received}\;\mathrm{mono}-I/O\;\mathrm{after}\;\mathrm{completing}\;\mathrm{chemoimmunotherapy}}$$

More specifically, the denominator was the number of patients who completed chemoimmunotherapy and subsequently switched to monotherapy for the same I/O during the study period. The numerator was the number of patients who completed chemoimmunotherapy with a shorter dosing schedule and switched to mono-I/O (the same I/O as that in the preceding chemoimmunotherapy regimen) with an extended dosing schedule.

## 3. Results

### 3.1. Study Attrition

Figure 1a–d show the study attrition overall (Figure 1a) and for each cohort (Figure 1b–d). A total of 7018 patients were selected who had been diagnosed with stage IV NSCLC, and three cohorts were selected from those patients. The cohort of patients with newly diagnosed stage IV NSCLC (*N* = 2579) had received a new diagnosis of stage IV NSCLC during either the pre-COVID-19 study period (February–July 2019) or the during- COVID-19 study period (February–July 2020) (Figure 1b). Another cohort of patients (*N* = 3519) had initiated or received at least one administration of an immunotherapy (i.e., pembrolizumab, nivolumab, atezolizumab) during either the pre-COVID or the during-COVID study period (Figure 1c). As for the final cohort of patients, 209 patients had completed chemoimmunotherapy and subsequently switched to monotherapy for the same I/O within the timeframes of interest (February–July 2019 and February–July 2020). Among these 209 patients, 8 were previously treated with a shorter dosing schedule and then switched to a long dosing schedule. All of those patients were captured during the February–July 2020 time period and treated with pembrolizumab (Figure 1d). These patients were designated as the switchers cohort.

### 3.2. Baseline Characteristics

Baseline characteristics were numerically similar across the cohorts (Table 1). Mean ages ranged from 67–70 years, the majority were White (ranging from 66–88%), and the majority were current or former smokers. At least half of each cohort had ECOG PS scores of 0–1, ranging from 50% for the switchers to 52% for the patients with stage IV NSCLC to 53% for the newly diagnosed with NSCLC to 57% for the new and continuing I/O patients. However, ECOG PS was not documented for approximately a third of patients in each cohort. Most patients (approximately two thirds) in each cohort had nonsquamous histology except in the switchers cohort, with 38%; however, the low sample size in the switcher cohort limits the drawing of conclusions.

Chi-square testing of independence was performed to compare gender, race, histology type, ECOG PS and type of treatment between the cohorts with stage IV NSCLC diagnosis before (*N* = 986) vs. during COVID-19 (*N* = 869) (Table 2). The timing cohorts were similar with respect to all characteristics except for ECOG PS (t(4) = 11.45, *p* = 0.022). According to an independent *t*-test, the pre-COVID-19 and during-COVID-19 cohorts had similar mean (SD) ages: 69.3 (10.3) years vs. 69.2 (10.2) years, t(1853) = −0.20 (*p* = 0.84).

### 3.3. Changes in Cancer Care Utilization before vs. during COVID-19 Timeframe

**Figure 2:**
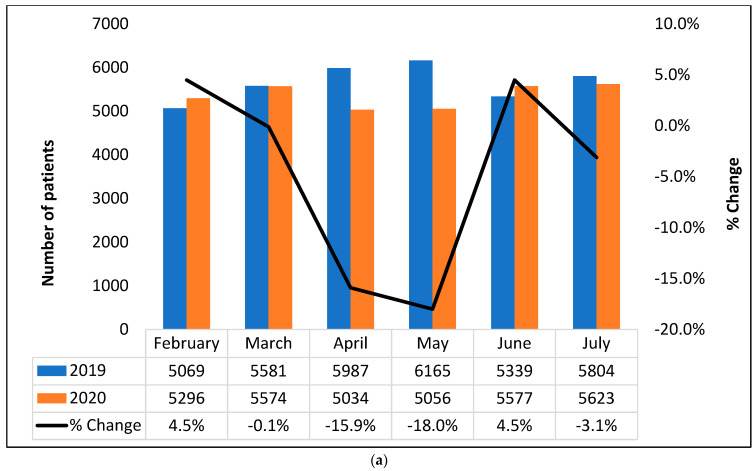

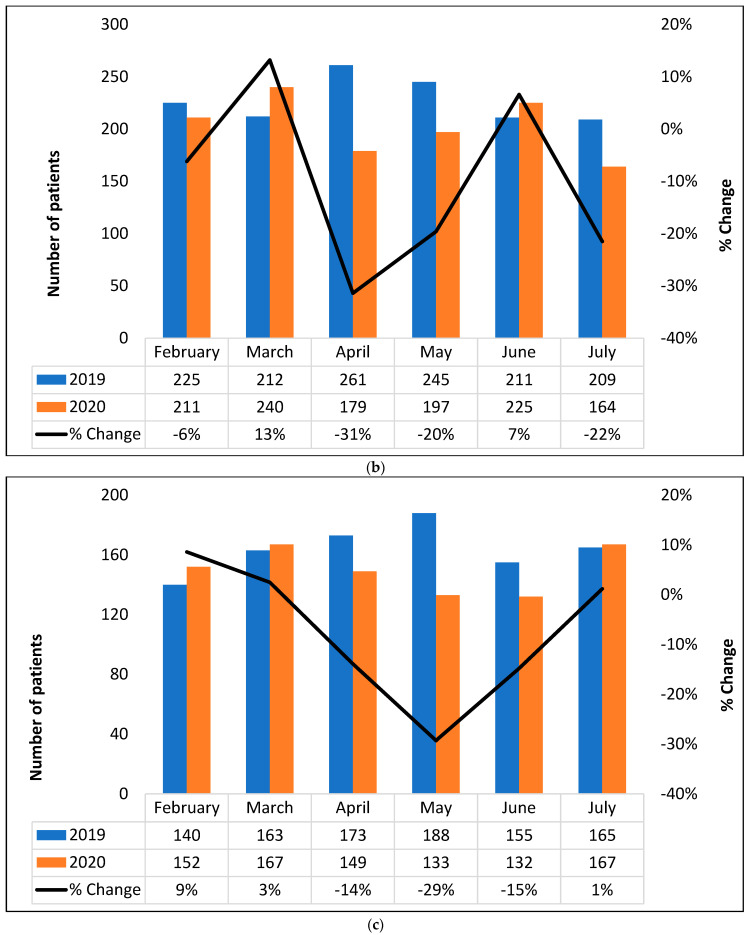
Effect of COVID-19 on patient encounters: comparing before and during COVID-19 (**a**) clinic/vital visits incidence (**b**) number of patients newly diagnosed with stage IV NSCLC (**c**) number of patients who initiated immunotherapy.

Based on month-to-month comparison, the numbers of patients with clinic/vital visits decreased significantly in April 2020 and May 2020 compared with 2019, by 15.9% and 18.0%, respectively (Figure 2a). A similar pattern was observed for the number of patients with newly diagnosed stage IV NSCLC and of patients who initiated immunotherapy, as they were also numerically lower in April 2020 and May 2020 compared with 2019 (Figure 2b,c). However, by June and July, the difference between before-COVID-19 and during-COVID-19 was markedly smaller.

### 3.4. Time to Treatment Initiation before vs. during COVID-19 Timeframe

The mean and median TTI were numerically shorter among patients diagnosed with stage IV NSCLC during the COVID-19 timeframe (32 and 26 days, respectively) compared with those diagnosed in the before-COVID-19 timeframe (36 and 29 days, respectively).

A multivariate linear regression model was run to find the association between the timing of NSCLC diagnosis and the TTI (Table 3). The regression model included the following variables: type of treatment, ECOG PS score, age, gender, race, and histology type, and was statistically significant (F(17) = 2.63, *p* = 0.0003).

On average, patients newly diagnosed with stage IV NSCLC in the during-COVID-19 timeframe vs. the before-COVID-19 timeframe had initiated treatment for NSCLC 4.6 days earlier (Least Squares Mean [LSM] = 24.1 vs. 28.7, respectively), after adjusting for type of treatment, ECOG PS score, age, gender, race, and histology type.

### 3.5. Delay in Immunotherapy Treatment before vs. during COVID-19 Timeframe

**Figure 3:**
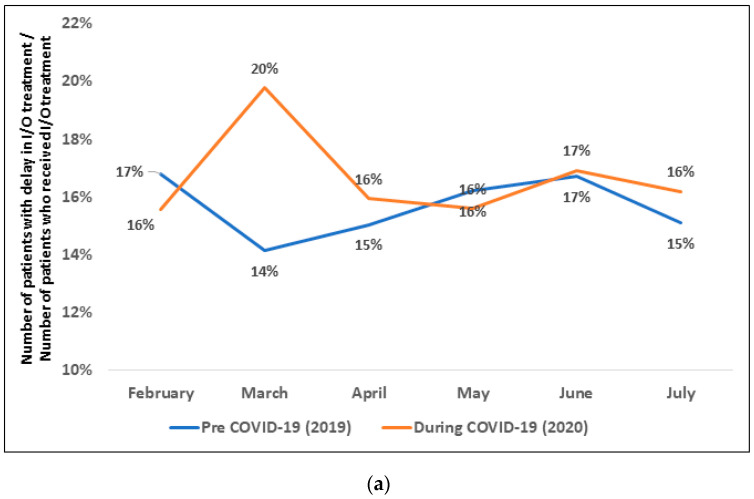

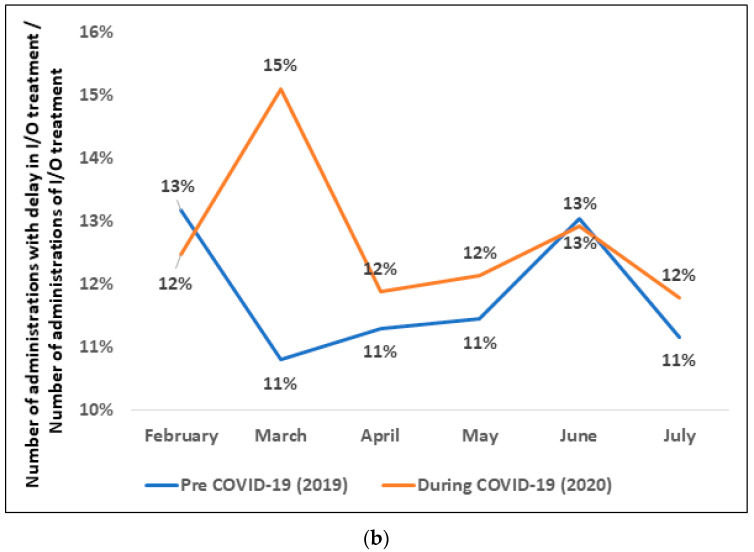
Delay in immunotherapy treatment in pre-COVID-19 and during-COVID-19 period (**a**) monthly percentage of patients who had a delay (**b**) monthly percentage of administrations that had a delay. I/O, immuno-oncology agent.

While there was a 3% decrease (17% to 14%) in patients with a delay in I/O treatment from February to March 2019 (pre-COVID-19 timeframe), there was a 4% increase (16–20%) from February to March 2020 (during-COVID-19 timeframe) (Figure 3a). Additionally, from February 2020 to March 2020, the number of patients with delay in I/O treatment increased by 31% (from 190 in February to 249 in March). When comparing month by month, 20% of patients had a delay in I/O treatment in March 2020 vs. 14% in March 2019, an increase of 6%. After May of both years, however, the differences between the pre-COVID-19 and during-COVID-19 percentages were less marked.

While there was a 2% decrease (13% to 11%) in administrations with delay in I/O treatment from February to March 2019, there was a 3% increase (12% to 15%) from February to March 2020 (Figure 3b). Additionally, from February 2020 to March 2020, the delay of administrations increased by 34% (from 191 in February to 255 in March). The percentage of administrations of delay in I/O in March 2020 was 15% vs. 11% in March 2019, an increase of 4%. After May of both years, however, the differences decreased between pre-COVID-19 and during-COVID-19.

### 3.6. Switching Rate from Shorter to Longer Immunotherapy Dosing Schedules before vs. during COVID-19 Timeframe

**Figure 4:**
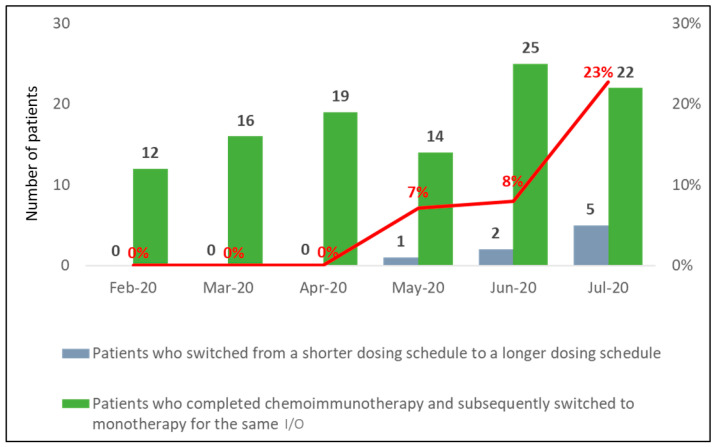
Overall switching rate for the immunotherapies of interest among patients with stage IV NSCLC; I/O, immuno-oncology agent.

The overall switching rate increased from 7% in May 2020 to 23% in July 2020 and this was attributed to pembrolizumab. The new approval of pembrolizumab 6W dosing schedule in late April 2020 provided a less frequent option to help patients reduce clinical visit frequency during the COVID-19 pandemic and resulted in a high switching rate from May 2020 onwards (Figure 4). There was no switcher identified in the study period of 2019.

## 4. Discussion

Changes in cancer care and immunotherapy utilization were found to be associated with the COVID-19 pandemic in this retrospective study in a large population of patients receiving community oncology-based care for stage IV NSCLC.

The impact of COVID-19 pandemic on healthcare delivery and access of care was considerable and immediate. Rapid decreases were observed in the numbers of patients with clinic or vital visits, initiation of I/O, and new diagnoses of stage IV NSCLC in April–May 2020 (during-COVID-19) compared with April–May 2019 (before-COVID-19). In addition, the rate of delays of I/O administrations and the proportions of patients delayed in receiving I/O treatment increased from February to March of 2020. These decreases and delays may have resulted from the considerable increase in COVID-19 cases during this timeframe and the endeavors to contain the pandemic through quarantine and lockdowns. However, when comparing the pre-COVID-19 and during-COVID-19 percentages for May and after, the differences in delay of I/O administrations became less marked. This may reflect the fact that during that time, most of the United States had begun easing the lockdowns [29].

Decreases in rates of clinic visits and diagnoses associated with the early COVID-19 period have been observed with other cancers in other real-world studies. A retrospective cohort study of 20 healthcare organizations showed approximate 50% decreases in patient encounters when comparing April 2020 vs. April 2019 for melanoma, breast cancer, lung cancer, hematologic, and colon cancer. In the same study, decreases in new diagnoses by 50% were also seen for all of these cancer types except for melanoma, with a 67% decrease [13]. Lou et al. performed a retrospective single-center analysis of patients with newly diagnosed lung, breast, or colorectal cancer [30]. Monthly rates of colonoscopies and mammographies were significantly lower during the second quarter of the pandemic vs. the pre-pandemic year, as were cancer diagnoses for lung, breast, and colorectal cancer. However, the rates increased again by the third quarter, as in our study [30]. Lee et al. conducted a retrospective study of colorectal cancer (CRC)-associated utilization within a large integrated healthcare organization covering over 4.5 million patients across Northern California [31]. They found reductions in rates of colonoscopies (diagnostic, surveillance, and screening) and of advanced adenoma and CRC detections when comparing April 2020 with April 2019. The difference decreased over the following months until November and December, when little difference between 2019 and 2020 remained. Colonoscopy procedure volume was 80% lower for April 2020 vs. April 2019 (2068 vs. 10,036 procedures, respectively). Notably, differences were the greatest with screening and surveillance compared with diagnostic colonoscopies (42%, 38%, and 20%, respectively). By September through December of 2020, the differences in total colonoscopy volume between 2020 and 2019 decreased to 4–10%. As in our study, they also observed reduction in diagnoses, specifically a 72% reduction in advanced adenoma detection rate (964 vs. 4603 patients with an adenoma per month, respectively) and a 44% reduction in colorectal cancer detection rate (55 vs. 98 patients with colorectal cancer per month, respectively) when comparing April 2020 with April 2019. The month-by-month differences between 2020 and 2019 decreased over the following months and disappeared by December 2020 [31]. This is in contrast with our study: while the difference decreased by June 2020, it increased again by July 2020. A longer study period in our study at least comparable to Lee et al. and Lou et al. would give more information on longer-term utilization patterns.

At the same time, in our study TTI did not increase among patients diagnosed with stage IV NSCLC during vs. before the COVID-19 timeframe. These findings suggest that patients with an advanced-stage cancer diagnosis are still inclined to start or resume treatment even with the limited resources and risk of COVID-19 infection. Parikh et al., in a study of a large EHR database, compared patients (*N* = 14,136) who had received a new diagnosis of metastatic (de novo or recurrent) solid tumor from January–July 2019 or from January–July 2020 [32]. As with our study, Parikh et al. also did not find a delay in systemic treatment initiation when comparing 2020 and 2019. At the same time, Parikh et al. found an approximate 5–6% increase in the proportion of de novo diagnoses of metastatic cancer during COVID-19 compared with pre-COVID-19 [32]. These results suggest that the need for prompt treatment initiation for metastatic disease overrode the pandemic-associated delays. Lou et al. found no difference in TTI for colorectal cancer or breast cancer, and in fact time to surgery was significantly shorter for CRC. For breast cancer, time to surgery and to chemotherapy were not significantly different; in fact, time to radiation and to endocrine therapy were significantly shorter during the pandemic. Use of I/O increased significantly in the pandemic vs. the pre-pandemic just for lung cancer but there was no significant change for breast or colon cancer. However, they did not specify the type, dosing or quarter, limiting conclusions about changes occurring early vs. later in the pandemic, types of I/O, and extended vs. standard dosing of I/O [30]. The results of Parikh et al. and Lou et al. suggest that pandemic-based delays in cancer screening and diagnoses may have led to a higher capacity at outpatient clinics and infusion centers for systemic treatment administration.

Neilson et al. analyzed prescription claims data for oral oncology medications from patients enrolled with a large national pharmacy benefits manager. They examined the changes from January–March (pre-period) to April–October (post-period) for 2019 and 2020. They then compared the two years. The number of new users of oral oncology medications per 100,000 members per month decreased by 12.7% from the pre period to the post period in 2020. From January-March to April-October of 2019, the decrease was 1.4%. The 2020 decrease was significantly longer (*p* = 0.048). Oral breast cancer medications drove most of that change; the difference between 2019 and 2020 was also significant (*p* = 0.040), but differences were not significant for leukemia, melanoma, lung or prostate cancer-specific oral medications [33].

In our study, the overall rate of switching from shorter to longer dosing schedules increased from May 2020 to July 2020. That profile fits the timing of the FDA approval of the pembrolizumab 6W dosing schedule in late April 2020 [21]. A similar pattern in the timeframe of treatment changes was seen in a prospective study at a single center examining patients with either NSCLC or SCLC. Treatment changes increased from March until the beginning of May of 2020, and then decreased by late May-early June 2020. Adopting the extended dosing schedules for pembrolizumab or durvalumab accounted for approximately 25% of the patients with treatment changes [4].

Other real-world studies have found adoption of extended dosing for other types of cancer for which extended dosing of ICI is indicated. Strohbehn et al. performed a retrospective analysis of the US Department of Veterans Affairs Corporate Data Warehouse, analyzing pembrolizumab administration data records from 1 April 2020 to 24 August 2021 for patients with primary tumor sites of lung, bladder, kidney, head and neck, melanoma, gastroesophageal, colorectal, and hepatocellular [34]. They found that adoption of extended dosing increased from April 2020 until it reached 32.6% by January 2021 and remained there through the end of the study period. Adoption of extended dosing beginning with the first pembrolizumab use reached its maximum in June 2020 and accounted for 20–25% of prescriptions starting in November 2020. Nearly all patients who initiated with extended dosing remained on that dose, while only 65% who initiated the standard dosing remained on that dose. Median time to discontinuation (TTD), which has been associated with overall survival and progression-free survival [35], was numerically but not significantly longer for all cancers in the extended dosing cohort (*N* = 159) vs. the standard interval dosing cohort (*N* = 676) (median follow-up 373 days; 168 days vs. 127.5 days; HR, 1.00; 95% CI, 1.00–1.00; *p* = 0.08). Among patients with NSCLC, median TTD for the extended (*N* = 53) vs. standard (*N* = 181) cohorts were 170 days and 112 days, respectively (median follow-up 377 days; HR, 1.00; 95% CI, 1.00–1.00; *p* = 0.15). However, comparison to our study is limited because the study populations are different. First, Strohbehn et al. had a single-payer population consisting of nearly all male veteran patients, and our study population includes a more diverse population in terms of sex and type of insurance. Second, our study included patients switching from chemoimmunotherapy while Strohbehn et al. excluded patients who also received chemotherapy. Finally, Strohbehn et al. had a longer study period, approximately a year longer than ours. Analyses through the end of 2021 and onwards will show cancer care patterns as the pandemic continues. Studies in a large and more diverse population, such as The US Oncology Network, are needed. Longer-term studies would also provide information on survival outcomes associated with the changes in cancer visits, diagnoses and treatment observed in this study.

Extended dosing schedules are also approved for nivolumab for colorectal cancer, malignant melanoma, and hematologic cancers [22]; and for atezolizumab for triple-negative breast cancer and small cell lung cancer [17]. Future studies exploring encounters, diagnosis, and changes in treatment for these therapies over the pre- and during-COVID time periods for these cancers in a single large population, such as The US Oncology Network, are needed.

### Strengths and Limitations

This retrospective analysis examined a population representing a considerable proportion of patients with stage IV NSCLC receiving community-based care in the United States.

However, there are also limitations that are characteristic of retrospective observational EHR studies. Data may be missing or incomplete. As iKM EHR data are recorded for clinical care and not for research, there may be data errors of omission and commission. For example, data for certain variables of interest were not documented for all study patients. If a patient received any care at a practice outside The US Oncology Network or the Onmark practices participating in the study, those data would not be available.

Generalizability of the study results may be limited. The location distribution of practices within this study, and the use of evidence-based guidelines within The US Oncology Network, may not reflect the general population of US patients with stage IV NSCLC. However, the iKM EHR data represents usual cancer care in a large network of community-based oncology practices.

## 5. Conclusions

This study provides insights into the impact on cancer care and treatment patterns for patients with stage IV NSCLC within the early stages of the COVID-19 pandemic. The study data show delays in initial cancer diagnoses, treatments, and patient visits in the earliest months within the pandemic period. These results show the considerable and immediate impact of the early stages of the COVID-19 pandemic on cancer care delivery. However, these delays were less pronounced later in the pandemic, and treatment initiations were not delayed when comparing the COVID-19 timeframe with the preceding year. The study results suggest that even with limited resources and lockdown conditions, treatment schedules were eventually adapted to provide patients with stage IV NSCLC in the United States with the best possible access to care. Future studies examining treatment and utilization patterns for other cancers and longer periods would give a more complete picture of how COVID-19 has been affecting cancer care, as well as the resulting long-term survival outcomes.

## Figures and Tables

**Table 1:** Baseline characteristics.

Baseline Characteristics	Stage IV NSCLC Patients (*N* = 7018)	Newly Diagnosed NSCLC Patients (*N* = 2579)	New and Continuing I/O Treated Patients (*N* = 3519)	Switchers (*N* = 8)
Gender—no. (%)				
Male	3512 (50)	1355 (53)	1890 (54)	6 (75)
Female	3501 (50)	1223 (47)	1628 (46)	2 (25)
Not documented	5 (0)	1 (0)	1 (0)	0 (0)
Age at Stage IV NSCLC diagnosis—years				
Mean (SD)	69 (10)	70 (10)	69 (10)	67 (7)
Race—no. (%)				
White	4887 (70)	1697 (66)	2531 (72)	7 (88)
Black or African American	652 (9)	237 (9)	338 (10)	0 (0)
Asian	222 (3)	67 (3)	73 (2)	1 (13)
Other	118 (2)	36 (1)	46 (1)	0 (0)
Not documented	1139 (16)	542 (21)	531 (15)	0 (0)
Region—no. (%)				
South	2744 (39)	1041 (40)	1441 (41)	4 (50)
Midwest	1919 (27)	715 (28)	977 (28)	1 (13)
West	1944 (28)	681 (26)	899 (25)	3 (38)
Northeast	410 (6)	141 (6)	202 (6)	0 (0)
Not documented	1 (0)	1 (0)	0 (0)	0 (0)
Histology Type—no. (%)				
Non-Squamous	4877 (69)	1761 (68)	2443 (69)	3 (38)
Squamous	1447 (21)	575 (22)	834 (24)	4 (50)
Not documented	694 (10)	243 (10)	242 (7)	1 (13)
ECOG PS score—no. (%)				
0	876 (13)	314 (12)	489 (14)	0 (0)
1	2761 (39)	1061 (41)	1516 (43)	4 (50)
2+	936 (13)	457 (18)	438 (12)	1 (13)
Not documented	2445 (35)	747 (29)	1076 (31)	3 (38)
Smoking history—no. (%)				
Former tobacco use	3937 (56)	1433 (56)	2091 (59)	5 (63)
Current tobacco use	1441 (20)	550 (21)	800 (23)	0 (0)
No history of tobacco use	1237 (18)	403 (16)	442 (13)	1 (13)
Not documented	403 (6)	193 (7)	186 (5)	2 (25)

ECOG PS, Eastern Cooperative Oncology Group performance status; I/O, immuno-oncology agent; NSCLC, non-small cell lung cancer.

**Table 2:** Factors associated with timing of stage IV NSCLC diagnosis: pre-COVID-19 vs. during-COVID-19.

Variable	Timing of Diagnosis			DF	t Value	*p* Value ^a^
	Total	Pre-COVID-19 (*N* = 986)	During-COVID-19 (*N* = 869)			
Gender, n				2	0.89	0.644
Female	890	472	418			
Male	964	513	451			
Not documented	1	1	0			
Race, n				4	7	0.136
White	1212	652	560			
Black	178	97	81			
Asian	54	31	23			
Other	27	19	8			
Not documented	384	187	197			
Histology, n				2	3.65	0.161
Nonsquamous	1339	728	611			
Squamous	416	204	212			
Not documented	100	54	46			
ECOG PS, n				4	11.45	0.022
0	266	150	116			
1	849	467	382			
2	240	133	107			
3	49	19	30			
Not documented	451	217	234			
Type of treatment, n				3	1.95	0.582
Chemo + I/O	937	513	424			
Chemo	258	133	125			
I/O	415	213	202			
Targeted therapy	245	127	118			

DF, degrees of freedom; ECOG PS, Eastern Cooperative Oncology Group performance status; I/O, immuno-oncology agent; NSCLC, non-small cell lung cancer. ^a^ Chi-square testing.

**Table 3:** Multivariate linear regression assessing association between timing of NSCLC diagnosis with time to treatment initiation.

Factor	Estimated Difference in Days	Standard Error	t Value	*p*-Value ^a^	Unadjusted R^2^ Value
Timing of diagnosis					0.008
Pre COVID-19	Reference	--	--	--	
During COVID-19	−4.6	1.29	−3.55	0.0004	
Type of treatment					0.013
I/O	Reference	--	--	--	
Targeted therapy	−9.7	2.28	−4.26	<0.0001	
Chemotherapy	−5.9	2.24	−2.64	0.0084	
Chemo + I/O combo	−6	1.64	−3.69	0.0002	
ECOG PS score					0.001
0	Reference	--	--	--	
1	1.9	1.93	0.97	0.3308	
2	2.3	2.45	0.95	0.3414	
3	−2.1	4.31	−0.48	0.633	
Not documented	1.7	2.11	0.81	0.4158	
Age (mean)	−0.1	0.06	−1.6	0.1108	0.035
Gender					0.001
Male	Reference	--	--	--	
Female	0.7	1.31	0.53	0.5958	
Not documented	−18.8	26.64	−0.71	0.4807	
Race					0.003
White	Reference	--	--	--	
Asian	−4.2	3.88	−1.08	0.2808	
Black	−2	2.31	−0.87	0.3827	
Other race	3.2	5.5	0.59	0.5562	
Not documented	−1.9	1.6	−1.2	0.2308	
Histology type					0.0004
Nonsquamous	Reference	--	--	--	
Squamous	−1.1	1.61	−0.65	0.5138	
Not documented	1.05	2.96	0.35	0.7235	

ECOG PS, Eastern Cooperative Oncology Group performance status; I/O, immuno-oncology agent; ^a^ ANOVA testing.

## Data Availability

The health data used to support the findings of this study are restricted by the US Oncology Institutional Review Board in order to protect patient privacy. For this reason, data used to support the findings of this study have not been made available.

## Supplementary Materials

The following supporting information can be downloaded at: https://www.mdpi.com/article/10.3390/curroncol30010059/s1, Supplementary Table S1: Dosing Schedules for the Immunotherapies of Interest in NSCLC.
