# Supplementary material for: Impact of COVID-19 Pandemic on Non-Small Cell Lung Cancer Care
Source: Curr Oncol. 2023 Jan 6;30(1):769–85. doi: 10.3390/curroncol30010059 (PMC9858327; doi:10.3390/curroncol30010059)
Supplement: Supplementary file 1 [file curroncol-30-00059-s001.zip › curroncol-2073186-supplementary.pdf]

Current Oncology

Supplementals

Impact of COVID-19 Pandemic on Non-Small Cell Lung Cancer Care

YiYuan Zhai †, Pooja Chopra †, David Kang, Nicholas J. Robert and Wei Zhang *

Table S1. Dosing Schedules for the Immunotherapies of Interest in NSCLC.

Drug	Dose schedule	Documented dose amount (mg)	Gap between infusions (days)
Nivolumab [22]	Q2W	240	12 to 18
	Q3W	360	19 to 25
	Q4W	480	26 to 32
	other[a]	other[a]	other[a]
Atezolizumab [17]	Q2W	840	12 to 18
	Q3W	1200	19 to 25
	Q4W	1680	26 to 32
	other[a]	other[a]	other[a]
Pembrolizumab [21]	Q3W	200	19 to 25
	Q6W	400	40 to 46
	other[a]	other[a]	other[a]

ICI, immune checkpoint inhibitor; Q2W, once every 2 weeks; Q3W, once every 3 weeks; Q4W, once every 4 weeks; Q6W, once every 6 weeks; [a]Other = any dose schedule, dose amount, or gap between infusions that was different from the treatment patterns recommended for each ICI.
