# Supplementary figures and images for: Impact of COVID-19 Pandemic on Non-Small Cell Lung Cancer Care
Source: Curr Oncol. 2023 Jan 6;30(1):769–85. doi: 10.3390/curroncol30010059 (PMC9858327; doi:10.3390/curroncol30010059)

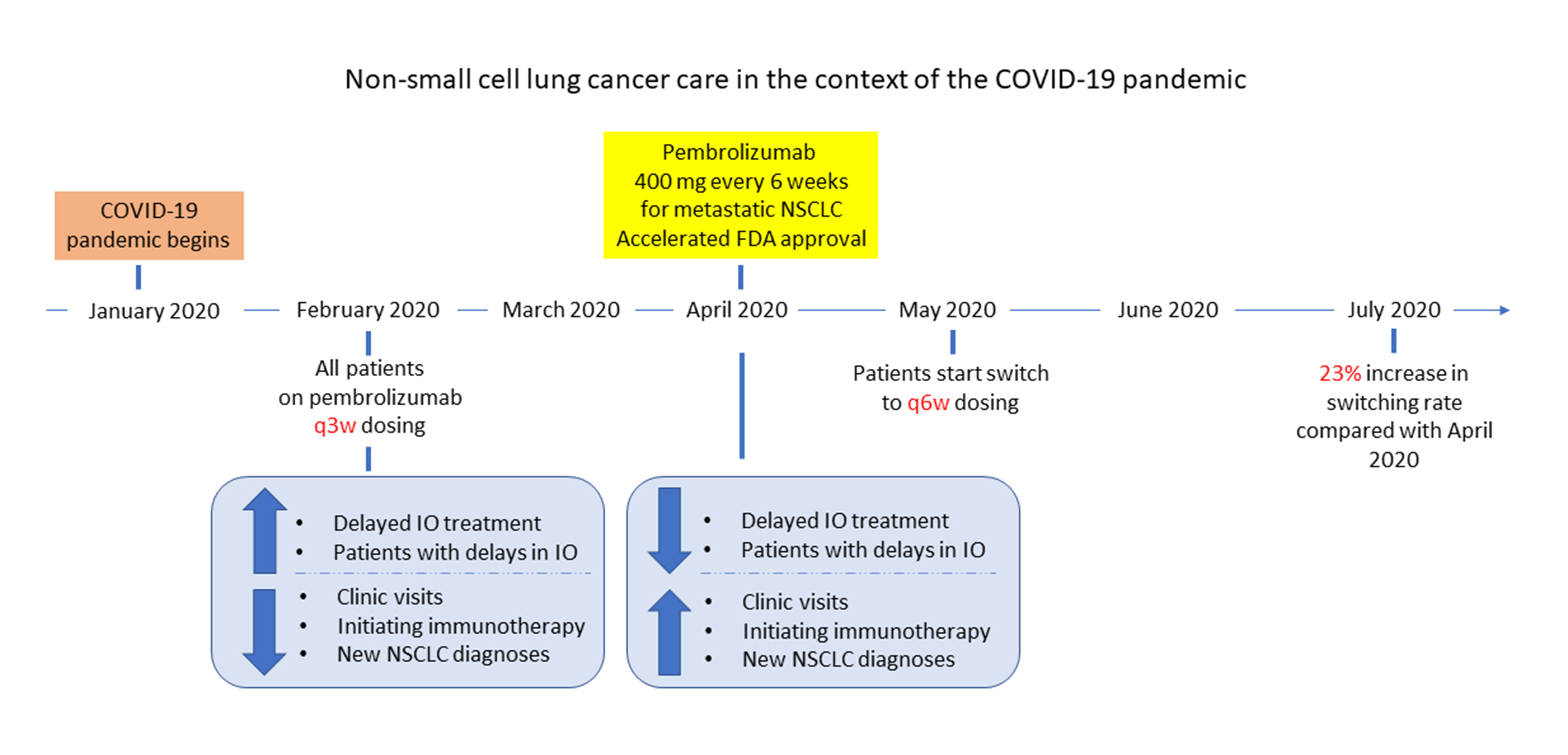

Supplement: Supplementary file 1 [file curroncol-30-00059-s001.zip › curroncol-2073186-graphical.jpg]
